# An ileo-ileal intussusception secondary to polypoid lipoma in a child, a case report and review of the literature

**DOI:** 10.1016/j.ijscr.2019.03.003

**Published:** 2019-03-20

**Authors:** Sarah Magdy Abdelmohsen, Mohamed Abdelkader Osman, Marwa T. Hussien

**Affiliations:** a Aswan University Hospital, Egypt; b Assiut University Hospital, Egypt; c South Egypt Cancer Institute, Assiut University, Egypt

**Keywords:** Small intestinal intussusception, Small intestinal lipoma, Unusual presentation of intussusception, Intramuscular lipoma

## Abstract

**Background:**

Intramuscular intestinal lipoma is one of the rare benign tumours of the small intestine in children and acts as a lead point of intussusception.

**Presentation of case:**

We describe a case in which a 4-year-old boy presented with recurrent attacks of colicky abdominal pain and non-bilious vomiting. Abdominal examination was irrelevant. Per rectum (PR) examination indicated an empty rectum without red currant jelly stool and non-palpable mass. Abdominal ultrasonography revealed a target sign of intussusception. After a period of conservative treatment, the child passed normal coloured and well-formed stool. Exploratory laparotomy referred to a long segment ileo-ileal intussusception with a polypoid lipoma as a lead point. Microscopic examination showed a benign non-capsulated intramuscular ileal lipoma.

**Discussion:**

Small bowel lipoma produces symptoms of intermittent bowel obstruction. Herein, the child presented with non- bilious vomiting, we attribute this to reflex sympathetic stimulation of the pylorus leading to pylorospasm.

**Conclusion:**

Small intestinal lipoma is a rare finding in children that may cause intussusception which does not resolve spontaneously.

## Highlights

• Small intestinal lipoma is a rare reason for ileoileal intussusception but should be included in the differential diagnosis for lead points causing intussusception in children.
• Dexamethasone as an adjuvant treatment may improve the outcome in cases of intussusceptions but this point needs future researches.
• Intermittent attacks of small intestinal intussusception may lead to dilation of the distal segment (Intussuscipiens). This mechanism may delay vascular compromise.
• High level of clinical suspicion needs to diagnose intussusception with unusual presentation.

## 1 Introduction

Most small bowel intussusceptions are transient, resolve spontaneously and only require sonographic follow-up a few hours later. Sometimes, persist due to an associated lead point. Our case had unusual symptoms and unusual lead point. This work has been reported in line with the SCARE criteria [1].

## 2 Case presentation

A 4-years-old boy was referred to our emergency department complained of recurrent attacks of colicky abdominal pain and non-bilious vomiting 5 times in the previous 24 h. The child had allergic rhinitis and recurrent attacks of gastroenteritis in the last four months.

Upon presentation to our hospital, the child was conscious, alert, comfortable in bed, and his vital signs were stable. Abdominal examination by inspection showed normal contour without abdominal distention and no bulging mass, with normal respiratory movement. By palpation, the abdomen was soft and lax, with no tenderness or rigidity and no palpable mass. PR examination revealed no palpable mass and an empty rectum without red currant jelly stool; the child had last defecated 12 h before.

Abdominal ultrasonography revealed a long segment small-intestinal intussusception extending from the subhepatic region through the right lumber region until the right iliac fossa region, which showed a pseudokidney appearance and no abdominal collection. Plain erect abdominal X-ray showed neither air-fluid levels nor gas under the diaphragm. Only fundic gas and a small amount of right colonic gas were observed. Abdominal computed tomography (CT) was not performed due to the poor resources of our hospital.

Our decision was to perform conservative treatment with multiple ultrasonographic examinations. The conservative approach was continued for 6 h duration with nothing per mouth (NPO). He had received medical treatment in the form of IV fluid, 3rd generation cephalosporin, ampicillin/sulbactam, metronidazole and antioedematous drugs such as dexamethasone, and lasix. During this period, the child had one attack of colicky abdominal pain and non-bilious gastric vomiting. However, during this period, the child passed well-formed normal brownish coloured stool. An abdominal examination had the same results as the previous clinical examination. PR examination revealed well formed, normal brown coloured stool without any redcurrant jelly secretion. Due to the recurrent symptoms of intestinal obstruction, the senior staff made a new decision to explore the abdomen.

**Fig. 1:**
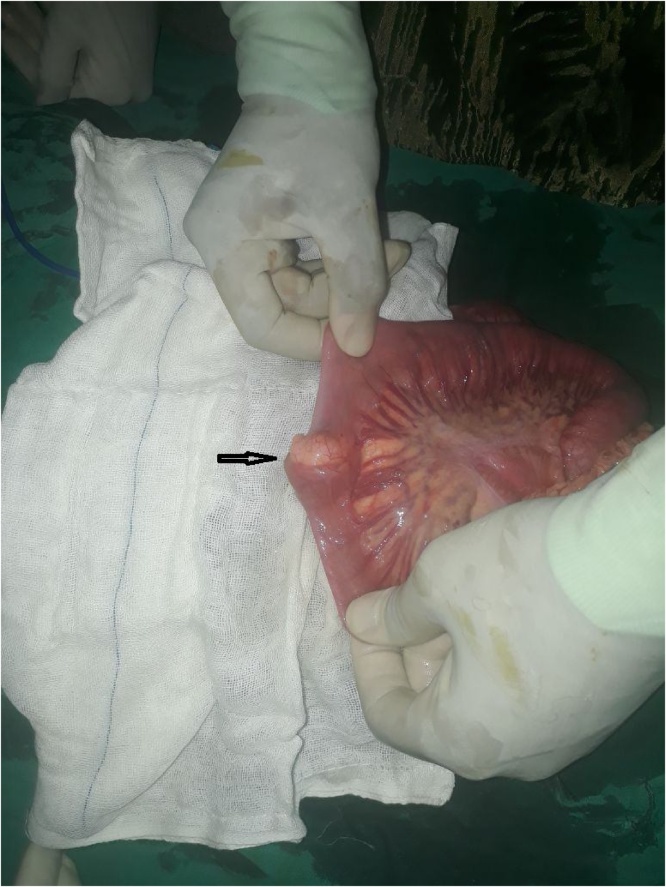
The lead point of the intussusception was a hemispherical mass, canary yellow in colour, 4 cm by 2 cm in diameter, firm, originating from the antimesenteric border of the ileum and not obstructing the lumen of the ileum.

After the child was administrated general anaesthesia, we palpated the abdomen and felt a hemispherical mass of 4 cm × 2 cm, movable in all directions without any restriction to its movement. A supraumbilical transverse incision was performed for exploration and revealed a long segment ileo-ileal intussusception approximately 50 cm long. Milking reduction was performed easily, without any intestinal ischaemia or intestinal wall oedema. The lead point of the intussusception was a hemispherical mass, canary yellow in colour and approximately 4 cm × 2 cm. It was firm, and rubbery inconsistency, did not obstruct the lumen of the ileum and originated from the antimesenteric border of the ileum. It was located 130 cm away from the ileocaecal junction (Fig. 1). Resection and anastomosis of the ileal segment containing the mass were performed with 2 cm safety margin on each side.

**Fig. 2:**
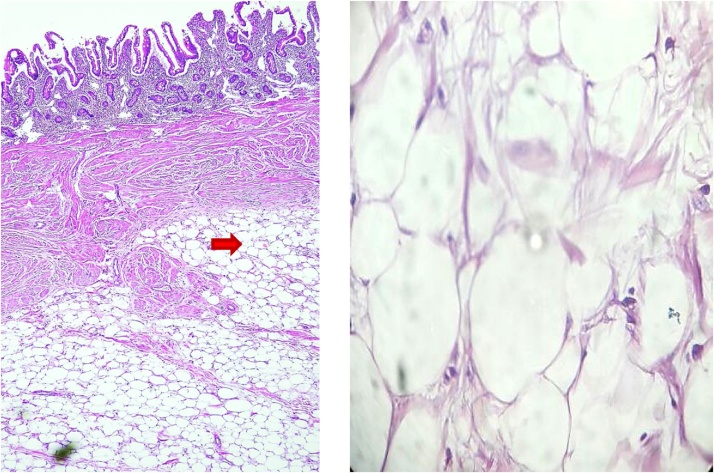
**Microscopic examination of small-intestinal lipoma:** (A) Low power examination showed lobules of fat separated by delicate fibrovascular trabeculae, dividing the musculosa (red arrow). (B) A high power examination showed mature fat cells with no malignant features.

A pathological macroscopic report described a polypoid submucosal fatty tumour 4 × 2 × 2 cm in size covered with intact mucosa. Microscopic examination showed a benign non-capsulated intramuscular soft tissue tumour formed of lobules of mature fat cells separated by delicate fibrovascular trabeculae (Fig. 2). The opposite small-intestinal mucosa was infiltrated by lymphocytes, plasma cells, and neutrophils. The final diagnosis was polypoid intramuscular lipoma of the ileum.

The postoperative period was uneventful, and the patient was discharged on the 7th postoperative day. At 6 months following surgery, he is free of symptoms, and no evidence of recurrence has been reported.

## 3 Discussion

The relative frequency of small-intestinal intussusception reported in the literature varies between 1.6% and 25% of all cases of intussusception [2]. The aetiology of intussusception is usually idiopathic or due to swollen mesenteric lymph nodes in patients in the typically affected age group. If recurrent intussusception occurs, or if intussusception occurs in older children, the presence of a pathological lead point must be considered [3].

Primary lipomas of the small intestine are rare mesenchymal neoplasms, representing 2.6% of nonmalignant tumours of the intestinal tract [4]. It arises mostly in an ileal submucosa, followed less frequently by intermuscular and serosal origins [5]. The peak age of incidence is in the 6th-7th decades of life and females seem to be more prone to lipomas [6]. Table 1 presents a review of the literature on pediatric small-intestinal lipoma [[7], [8], [9]].

**Table 1:** The characteristics of the reported cases of pediatric small intestinal intussusception induced by a lipoma [[7], [8], [9]].

case	Age	Gender	Diagnostic modality	Tumor location	References
1	14ys	Male	CT	Ileocolic with 3 discrete submucosal lipomas measuring 2 to 2.5 cm in the greatest dimension	[7]
2	7ys	Male	CT	In the ileum, 50 cm from the ileocolic valve on the oral side.	[8]
3	12ys	Male	CT	a single multilobulated duodenal lipoma	[9]

Small bowel lipoma produces symptoms of intermittent bowel obstruction. Very small ones may be asymptomatic [5]. Herein, in spite of small size lipoma, the child presented with non- bilious vomiting. We attribute this to reflex sympathetic stimulation of the pylorus leading to pylorospasm. At laparotomy, we discovered a long segment ileo-ileal intussusception without ischaemic compromise or wall oedema, even the child passed one-time well-formed stool. We attribute this phenomenon to intermittent attacks of intussusception lead to dilation of the distal segment (Intussuscipiens), so, vascular compromise does not occur. We believe in the rule of dexamethasone as an adjuvant treatment to decrease bowel wall oedema and improve the outcome. Gluckman, et al., conclude by the effectiveness of Dexamethasone as an adjuvant in reducing intussusception recurrence rates [10]. This point needs to be addressed in future studies as it is deficient.

## 4 Conclusion

Considering the rarity of small-intestinal intramuscular lipoma in children it should be included in the differential diagnosis of abdominal pain of doubtful origin.

## Conflicts of interest

No conflict of interest exists for any of the authors in this case report.

## Funding

None.

## Ethical approval

The institutional Ethics committee (Aswan faculty of a medical ethical committee) had reviewed and approved my case report. EC Ref NO: asw327219.

## Consent

Written informed consent was obtained from the patient’s legal guardian(s) for publication of this case report and any accompanying images. A copy of the written consent is available for review by the Editor-in-Chief of this journal.

## Author contribution

Sarah Magdy Abdelmohsen, the main operator of the case study, was responsible for data collection, analysis, and the writing of the article and is the "Corresponding author".

Mohammed Abdelkader Osman was the main supervisor, and reviewer of the paper.

Marrow Tammam Hussein was the pathologist performed the microscopic photography.

## Registration of research studies

I do not make any register, it is a case report.

## Guarantor

Sarah Magdy Abdelmohsen.

## Availability of data and materials

All data and materials are available in case of request.

## Provenance and peer review

Not commissioned externally peer reviewed.
